# Impact of a Mobile Application for Tracking Nausea and Vomiting During Pregnancy (NVP) on NVP Symptoms, Quality of Life, and Decisional Conflict Regarding NVP Treatments: MinSafeStart Randomized Controlled Trial

**DOI:** 10.2196/36226

**Published:** 2022-07-05

**Authors:** Elin Ngo, Maria Bich-Thuy Truong, David Wright, Hedvig Nordeng

**Affiliations:** 1 PharmacoEpidemiology and Drug Safety Research Group Department of Pharmacy University of Oslo Oslo Norway; 2 School of Allied Health Professions University of Leicester England United Kingdom; 3 Centre for Pharmacy University of Bergen Bergen Norway; 4 Department of Child Health and Development National Institute of Public Health Oslo Norway

**Keywords:** eHealth, mHealth, decision support tool, nausea and vomiting, pregnancy, RCT

## Abstract

**Background:**

Pregnant women are active users of mobile apps for health purposes. These apps may improve self-management of health-related conditions. Up to 70% of pregnant women experience nausea and vomiting (NVP). Even mild NVP can significantly reduce quality of life (QoL), and it can become an economic burden for both the woman and society. NVP often occurs before the first maternal care visit; therefore, apps can potentially play an important role in empowering pregnant women to recognize, manage, and seek appropriate treatment for NVP, when required.

**Objective:**

This study investigated whether the MinSafeStart (MSS) mobile app could impact NVP-related symptoms, QoL, and decisional conflict regarding NVP treatment.

**Methods:**

This randomized controlled trial enrolled 268 pregnant women with NVP in Norway from 2019 to 2020. The intervention group had access to the MSS app, which could be used to track NVP symptoms and access tailored advice. NVP severity was rated with the Pregnancy Unique Quantification of Emesis (PUQE) score. The control group followed standard maternal care. We collected data on maternal baseline characteristics, NVP severity, QoL, and decisional conflict using 2 sets of online questionnaires. One set of questionnaires was completed at enrollment, and the other was completed after 2 weeks. We performed linear regression analyses to explore whether the use of the MSS app was associated with NVP severity, QoL, or decisional conflict.

**Results:**

Among the 268 women enrolled in the study, 192 (86.5%) completed the baseline questionnaires and were randomized to either the intervention (n=89) or control group (n=103). In the intervention group, 88 women downloaded the app, and 468 logs were recorded. In both groups, women were enrolled at a median of 8 gestational weeks. At baseline, the average PUQE scores were 4.9 and 4.7; the average QoL scores were 146 and 149; and the average DCS scores were 40 and 43 in the intervention and control groups, respectively. The app had no impact on NVP severity (aβ 0.6, 95% Cl −0.1 to 1.2), QoL (aβ −5.3, 95% Cl −12.5 to 1.9), or decisional conflict regarding NVP treatment (aβ −1.1, 95% Cl −6.2 to 4.2), compared with standard care.

**Conclusions:**

Tracking NVP symptoms with the MSS app was not associated with improvements in NVP symptoms, QoL, or decisional conflict after 2 weeks, compared with standard care. Future studies should include a process evaluation to improve our understanding of how pregnant women use the app and how to optimize its utility within maternity care. Specifically, studies should focus on how digital tools might facilitate counseling and communication between pregnant women and health care providers regarding NVP management during pregnancy.

**Trial Registration:**

ClinicalTrails.gov (NCT04719286): https://www.clinicaltrials.gov/ct2/show/NCT04719286

## Introduction

### Background

Pregnant women and women of reproductive age are active users of mobile apps for health purposes [1]. Available apps are designed for promoting self-management of chronic diseases, such as migraine and diabetes; tracking gestational weeks, weight, and belly measurements during pregnancy; and keeping track of pregnancy development in general [1,2]. These apps are often used to supplement routine care, because women tend to search for health-related information early in pregnancy, before and after health consultations, and when making decisions [1,3-5]. Often, the primary motivation for using apps is the need for easily accessible health information [6]. Our recent systematic review on decision support tools in pregnancy revealed that few studies had investigated the effect of digital tools on the course of pregnancy and pregnancy-related ailments. However, available studies have shown that apps could have a positive impact on the knowledge level of pregnant women, when integrated as part of patient care. Pregnant women also seemed to appreciate and were satisfied with digital tools [7].

Nausea and vomiting in pregnancy (NVP) is one of the most common pregnancy-related conditions. NVP affects up to 70% of pregnant women worldwide [8,9]. NVP symptoms often occur during the first few weeks of pregnancy, on average, at around gestational week 4 [10]. The etiology of NVP is not clearly understood, but it is thought to be multifactorial and complex [10]. The severity of NVP can range from mildly uncomfortable to hyperemesis gravidarum (HG), which is the most severe form of NVP. HG affects 1%-3% of all pregnant women, and it is the most common reason for hospitalization in early pregnancy [8]. Although HG is a relatively rare condition, it is essential to recognize the burden of NVP in general. Previous studies have shown that even mild NVP symptoms significantly reduce quality of life (QoL) of pregnant women and their willingness to become pregnant again [11,12]. Moreover, as the severity of NVP increases, the costs for society increase due to increased hospital and emergency room admissions, health care visits, prescribed medications, and income loss for both the woman and her partner [13].

NVP treatment guidelines recommend early recognition and treatment to prevent or reduce more severe symptoms. The first-line management of mild symptoms consists of nonpharmacologic measures, including lifestyle and dietary changes (Multimedia Appendix 1). Pharmacological treatment is indicated when NVP symptoms are moderate to severe or when symptoms significantly impact the women’s daily activities [14,15]. The first NVP symptoms typically occur early in pregnancy and, often, before the first maternal care visit. Therefore, it is important to empower pregnant women to ensure that they can optimally manage NVP symptoms [15,16].

Digitalization, eHealth initiatives, and the wide use of the internet have opened up new possibilities for using digital tools in maternal care [17]. Mobile apps can enable pregnant women to take a more active role in self-care and disease management during pregnancy. Moreover, these apps can provide large amounts of patient-generated data during pregnancy for research purposes [17,18]. The Pregnancy Unique Quantification of Emesis (PUQE) score is an internationally validated tool for categorizing the severity of NVP based on 3 questions regarding vomiting, nausea, and retching symptoms [19,20]. In the latest (2009) version of the PUQE score, women are asked to rate the severity of symptoms that occurred in the last 24 hours [19]. A translated and validated Norwegian version of the PUQE score became available in 2015 [21]. Incorporating the PUQE score into an app could potentially empower women by improving their management of NVP. The app could allow women to track symptoms over time and record responses to interventions. Because 99%-100% of women of reproductive age use smartphones [22] and most women use health-related apps [23,24], digital tools should be particularly suitable for maternal care.

A recent review pointed out that, although there is a growing number of apps available for monitoring and managing health-related issues, the majority are never tested nor clinically validated [25]. That finding implied that it remains largely unknown whether available apps are beneficial or whether they even have an effect on clinical outcomes. A prior study showed that integrating apps into professional clinical services could potentially improve the effectiveness of health care [26]. Our previous review concluded that the innovative use of eHealth initiatives and digitalization could potentially empower pregnant patients and improve maternal care [7]. However, at the same time, a more scientific approach is needed for testing and evaluating these apps and other digital tools. Indeed, health care providers should encourage patients to use only tools that are beneficial and effective as a supplement to routine maternity care.

### Objective

The primary aim of this study was to investigate whether the MinSafeStart (MSS) mobile app could impact NVP severity in pregnant women. The secondary aims were to assess whether the MSS app could affect the QoL of pregnant women and improve their ability to make decisions regarding NVP treatment.

Specifically, the primary research question was: Will women who use the MSS app for 2 weeks have different NVP symptoms, based on PUQE scores, compared with women who follow standard maternal care without the MSS app?

The specific secondary research questions were: (1) Will women who use the MSS app for 2 weeks have different QoL, based on Health-related Quality of Life for Nausea and Vomiting during Pregnancy (NVPQOL) scores, compared with women who follow standard maternal care without the MSS app? (2) Will women who use the MSS app for 2 weeks have different decisional conflict scale (DCS) scores regarding NVP treatment, compared with women who follow standard maternal care without the MSS app? (3) Will the use of the MSS app modify the association between the PUQE score and the NVPQOL score (ie, is the MSS app an effect modifier)?

## Methods

### Study Design, Study Population, Recruitment, and Sample Size

The MinSafeStart study was a randomized controlled trial. We recruited pregnant women in Norway between September 2019 and June 2020. All pregnant women over 18 years old who were currently experiencing NVP, owned a smartphone (iOS or Android), and could speak and understand Norwegian were eligible for inclusion.

Participants were primarily recruited through social media advertisements. Invitations to participate in the study were available on the study Facebook page, the Norwegian Hyperemesis Gravidarum Patient Organization’s Facebook page, and other pregnancy-related web pages or forums, such as “altformamma.no” (all for mommy) and “tryggmammamedisin.no” (safe mother medications). Invitations were additionally accessible through the Helseoversikt app. Helseoversikt is a digital platform used by health care centers all over Norway that provides relevant health information to pregnant women and parents.

All invitations to participate contained a link to the online consent form. When the women signed the consent form and responded to the baseline questionnaire, they were automatically randomized to either the intervention or control group. Both groups received emails with information about the study group to which they were assigned. The intervention group also received an email with instructions on how to download and use the app.

Results from the power analysis suggested that we would need a total of 250 pregnant women (n=125 in each group, 2-tailed hypothesis) to detect a mean difference of 3 points in the PUQE score between the groups, with a power of 80% (Cohen d=0.5). This total sample size included a 25% dropout rate.

### Randomization

An automated software program was specifically developed for the project. The software automatically managed participant enrollment, randomization to study groups, and email distributions of electronic information and online questionnaires to the study participants. This software was developed for the project by the University Center for Information Technology (USIT) at the University of Oslo.

### Development of the MinSafeStart Mobile Application

**Figure 1:**
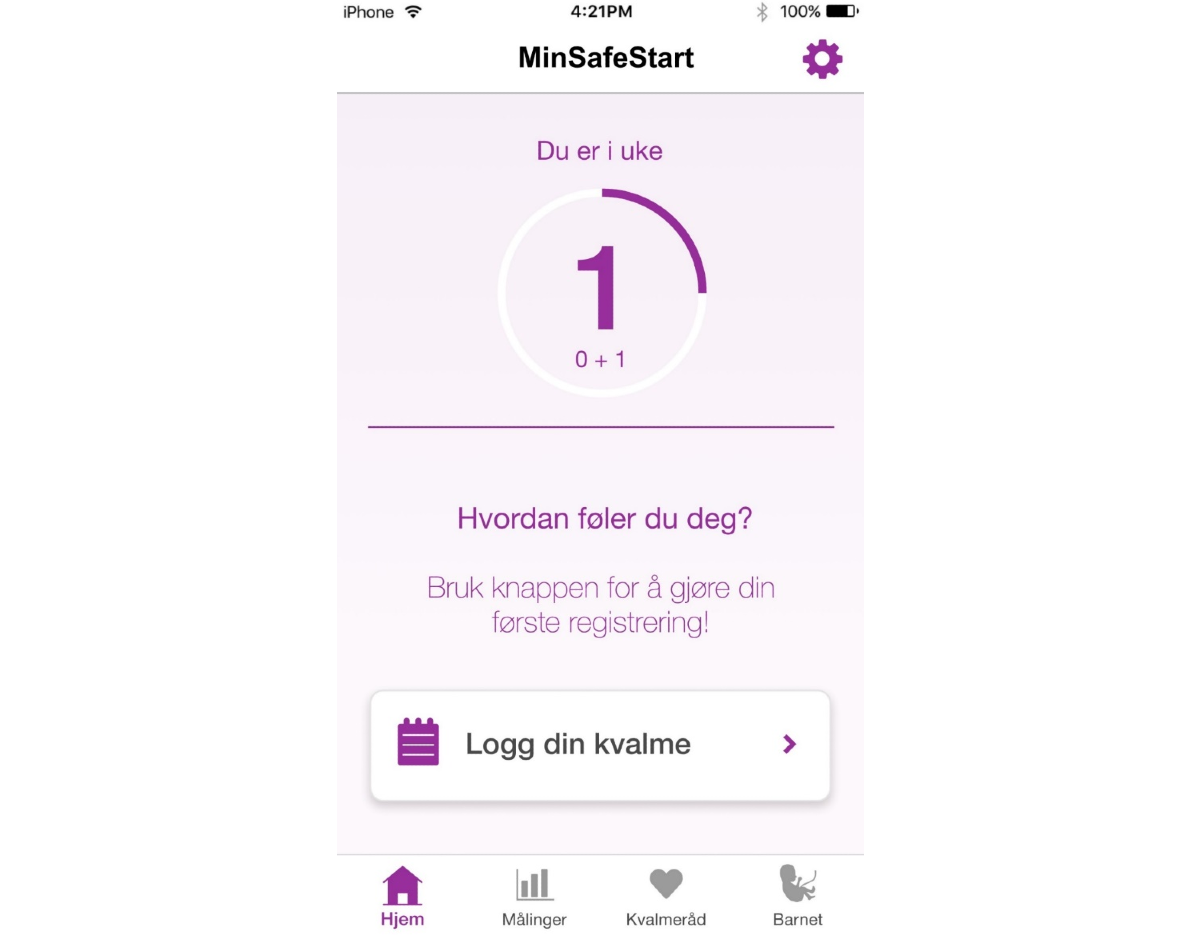
Front page of the MinSafeStart application (in Norwegian) for pregnant women to track nausea and vomiting, showing the user's gestational week at the top, text in the center (“How do you feel? Use the button below to log your NVP symptoms”), and button to log nausea and vomiting in pregnancy (NVP) symptoms.

**Figure 2:**
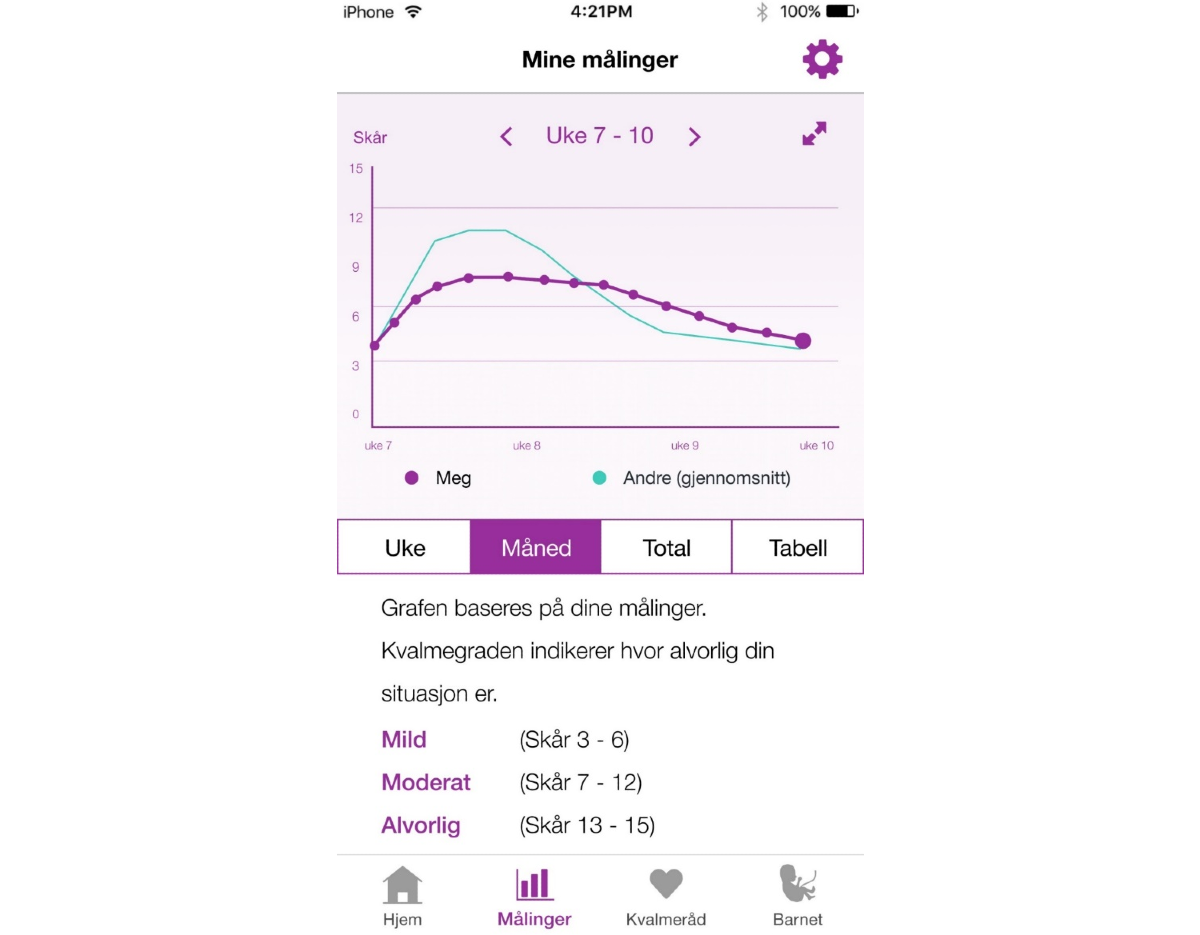
The MinSafeStart app (in Norwegian) for pregnant women with nausea and vomiting (NVP) shows the women’s NVP loggings (Mine Malinger) as the user’s NVP scores (purple) as a graph over time (week [Uke], month [Måned], for all data recorded in the app [Total]), compared with the mean Pregnancy Unique Quantification of Emesis (PUQE) score of other pregnant women (blue line), or as a table (Tabell). The bottom section shows the numeric rating scale for NVP symptoms. Alvorlig: severe; Moderat: moderate; Skår: Score.

The MSS app was a patient-centered app for women with NVP. Our research group developed the MSS app in collaboration with interaction designers, programmers, and researchers from USIT. The app utilized the daily PUQE score (Multimedia Appendix 2) to categorize NVP severity (ie, mild, moderate, or severe), and it displayed the fluctuations over time in a graph (Figures 1 and 2). The aim of the app was to assist pregnant women in identifying and managing NVP. The app tracked their NVP symptoms every day and provided tailored advice according to the severity of their symptoms. All women with NVP symptoms received lifestyle and dietary advice (eg, stay hydrated, eat small meals frequently, and get some rest). Women that experienced severe NVP also received information about medical treatments. The app alerted the woman to seek appropriate treatment when she logged PUQE scores >13 for more than 3 consecutive days. The app was user tested in July 2018. The user test included 9 women who completed a structured interview with a set of tasks and questions regarding the app. Of these 9 women, 5 also participated in a focus group to discuss and share their experiences and opinions about the app. The user test results showed that the app was user-friendly and had the potential to empower women who experienced NVP to improve their management skills and treatment decisions. Nevertheless, some minor issues were mentioned in the user test and focus group that could be improved (ie, explanations of terminologies, an opportunity to change the due date, links to external information, an overview of previously logged scores, and the layout and design). These suggestions were incorporated into the app to make it as user-friendly as possible before it was launched for iOS and Android smartphones.

### Data Collection

In this MinSafeStart study, we collected data from the MSS app and from 4 sets of questionnaires (Q1-Q4) that were completed electronically. Q1 was administered to participants at enrollment (baseline), and Q2 was administered 2 weeks later. Q3 and Q4 were additional follow-up questionnaires administered at 4 weeks and 6 weeks after baseline, respectively. All questionnaires were sent to participants by email with the automated software developed for the study. This study only analyzed data from the Q1 and Q2 sets of questionnaires. We selected a 2-week follow-up for this study because we considered that 2 weeks were sufficient to become familiar with the app.

All data collected from the app and questionnaires were automatically encrypted and stored at the Service for Sensitive Data at the University of Oslo (TSD). The TSD platform is available to collect, store, and analyze sensitive data [27]. The platform is protected by a 2-step password system and meets all the necessary requirements to maintain compliance with Norwegian regulations regarding individual privacy. The data are not accessible outside of the TSD. Only registered researchers within the project had access to the data and the encryption key.

The study is reported in accordance with the CONSORT-EHEALTH checklist (Multimedia Appendix 3).

### Intervention Group

All women in the intervention group were given access to the MSS app in addition to standard maternal care. They were free to log their NVP symptoms into the app whenever convenient. Standard maternity care in Norway is free of charge. It includes 9 routine checkups with a midwife or physician and 1 ultrasound scan at gestational week 18 [28].

The app recommended logging symptoms every 24 hours because the PUQE score was calculated based on NVP symptoms over the past 24 hours. Users could also compare their symptoms to the expected population average NVP score. Thus, women received individual treatment advice based on their PUQE scores (Multimedia Appendix 1). Women also received general dietary and lifestyle advice (eg, get some rest, stay hydrated, eat small meals frequently, and avoid fatty and spicy foods [29]) independent of their PUQE score. Women with moderate or severe symptoms received additional advice about antiemetic medications. When a woman scored ≥13 points (ie, severe NVP) for more than 3 consecutive days, she would see a pop-up message that encouraged her to see the doctor.

### Control Group

The control group received only standard maternal care.

### Outcome Measures

#### NVP Severity

The PUQE score was internationally validated for rating the severity of NVP symptoms over the past 24 hours (Multimedia Appendix 2) [19,21]. The scale consists of 3 questions. Each question is rated from 1 to 5. The total score ranges from 3 to 15 points, where ≤6 points indicate mild NVP, 7-12 points indicate moderate NVP, and 13 or more points indicate severe NVP. This study utilized the translated and validated Norwegian version of the PUQE [21]. We evaluated the change in PUQE scores from Q1 to Q2 (ie, after 2 weeks).

#### Quality of Life

The NVPQOL was used to rate QoL [30] over the past week (Multimedia Appendix 2). The score includes 30 items covering 4 general domains: physical symptoms and aggravating factors, fatigue, emotions, and limitations. Each item is rated on a Likert scale that ranges from 1 (never) to 7 (all the time). The total score ranges from 30 to 210 points, and lower scores indicate a better QoL. The NVPQOL score is significantly associated with the SF-12 health-related QoL questionnaire [30]. We evaluated the change in NVPQOL scores from Q1 to Q2.

#### Decisional Conflict

Decisional conflict was measured with the decisional conflict scale (DCS). The DCS measures the individual’s perception of uncertainty in choosing options, modifiable factors that contributed to uncertainty, and decision-making effectiveness [31,32] (Multimedia Appendix 2). The DCS has been widely used in previous studies among pregnant women to evaluate their decision-making abilities regarding the use of antidepressants and the choice between vaginal birth or cesarean section [33,34]. The DCS consists of 16 items and 5 response categories (strongly agree, agree, neither agree nor disagree, disagree, and strongly disagree). The total score ranges from 0 to 100 points. Scores below 25 points indicate low decisional conflict, scores of 25 to 37.5 points indicate moderate decisional conflict, and scores above 37.5 points indicate high decisional conflict. We evaluated the change in DCS scores from Q1 to Q2.

### Statistical Analyses

#### Descriptive Analysis

Categorical variables (ie, relationship status, education level, work situation, parity, and prior NVP symptoms) are presented as percentages for each group (intervention and control groups). Continuous variables are presented as the median and range (eg, gestational week) or the mean and SD (eg, maternal age). We performed a Pearson Chi-squared test to compare categorical variables, except when the expected cell count was less than 5; in those cases, we performed a Fisher exact test. We performed a Student *t* test to compare continuous variables. All analyses were performed with Stata/MP v.16.1. *P* values <.05 were considered statistically significant.

#### Primary and Secondary Analyses

We performed univariate and multivariable linear regression analyses to estimate associations between the use of the MSS app and (1) NVP severity, (2) QoL, and (3) decisional conflict. All results are presented as the crude and adjusted beta-coefficients (β) with 95% CIs. We adjusted the multivariable linear regression model with predefined covariates (ie, baseline PUQE score, baseline NVPQOL score, and baseline DCS) [35].

#### Subanalyses

We performed a prespecified stratified analysis to assess whether employment in the health sector modified the association between the use of the MSS app and the PUQE score. We reasoned that women employed in the health sector might have better access to information and advice regarding NVP management, and thus, they may have less need for an app to track their NVP symptoms, compared with women employed in other settings. Alternatively, they may have received more support or information from co-workers in the field that allowed them to capitalize on the information provided by the app, compared with women employed in other settings.

### Ethical Approval

This study was approved by the Regional Committees for Medical and Health Research Ethics in Norway (Ref: 2018/2298). Informed consent to participate in the study was obtained from all participants.

## Results

### Study Population

**Figure 3:**
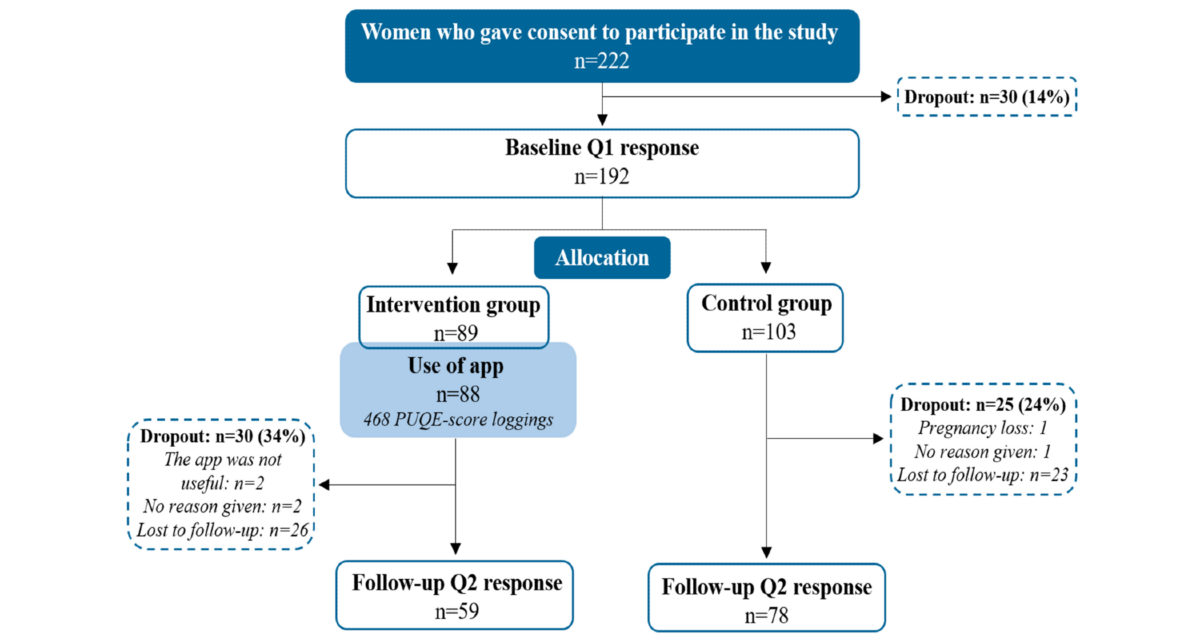
Flowchart of the study participants in the enrolled group, allocation groups, and follow-up groups. app: MinSafeStart mobile app; PUQE: Pregnancy Unique Quantification of Emesis; Q1: Questionnaire 1.

Overall, 268 women consented to participate in the study (Figure 3). Of these, 192 (86.5%) responded to the baseline questionnaires (Q1) and were randomized to either the intervention group (n=89) or the control group (n=103). In total, 137 women responded to the follow-up questionnaires 2 weeks later (Q2). The dropout rates were 34% (30/89) for the intervention group and 24.3% (25/103) for the control group. The main reason for dropout was “lack of response.”

At enrollment, the median stage of pregnancy was the same in both groups: 8 (range 4-36) gestational weeks in the intervention group and 8 (range 4-39) gestational weeks in the control group. These groups had the same mean age at enrollment: 32 (SD 4.6) years and 32 (SD 3.9) years, respectively. Most women had been pregnant previously (65/89, 73%, and 76/103, 73.8%, respectively). In both groups, 80% (52/89 and 61/103, respectively) had experienced NVP in at least one previous pregnancy. None of the women reported severe NVP (ie, PUQE score ≥13) at baseline. A comparison of baseline characteristics using the Student *t* test, Chi-squared test, or Fisher exact test indicated no statistical difference (all *P*<.05) between the 2 study groups (Table 1).

**Table 1:** Baseline characteristics of the study population (n=192), stratified by whether they used the MinSafeStart (MSS) app (intervention) or received standard maternity care (control).

Characteristics			Intervention group (n=89)		Control group (n=103)
Gestational week at enrollment, median (range)			8 (4-36)		8 (4-39)
Age (years), mean (SD)			32 (4.6)		32 (3.9)
**Relationship status, n (%)**					
	Married/cohabitation	85 (95.5)		100 (97.1)	
	Other^a^	4 (4.5)		3 (2.9)	
**Higher education, n (%)**					
	Yes	69 (77.5)		85 (82.5)	
	No	20 (22.5)		18 (17.5)	
**Working situation, n (%)**					
	Employed	55 (61.8)		60 (58.2)	
	Employed in the health sector	19 (21.4)		31 (30.1)	
	Other^b^	15 (16.8)		12 (11.7)	
**Primigravida, n (%)**					
	Yes	24 (27.0)		27 (26.2)	
	No	65 (73.0)		76 (73.8)	
**NVP^c^ during previous pregnancy/pregnancies, n (%)**					
	Yes	52 (80.0)		61 (80.3)	
	No	13 (20.0)		15 (19.7)	

^a^Includes single/unmarried and divorced/separated women.

^b^Includes students and unemployed women.

^c^NVP: nausea and vomiting during pregnancy.

### The Intervention

Of the 89 women randomized to the intervention group, 88 downloaded the MSS app. These women performed a total of 468 logs. Because they were not satisfied with the app, 2 women dropped out of the study. They reported no benefit in using the MSS app.

### Impact on NVP Severity

The groups showed no differences in the change in PUQE scores between Q1 and Q2 (adjusted β 0.6, 95% Cl −0.1 to 1.2). Among women employed in the health sector, those who used the MSS app had a significantly higher PUQE score (adjusted β 2.1, 95% Cl 0.9 to 3.2) after 2 weeks than those who did not use the app. However, among women employed in other sectors, the PUQE scores were not significantly different between the intervention and control groups (Table 2).

**Table 2:** Associations between the use of the MinSafeStart (MSS) app and the Pregnancy Unique Quantification of Emesis (PUQE) score.

Analysis		Baseline (Q1) PUQE score^a^, mean (SD)	Follow-up (Q2) PUQE score, mean (SD)	Change in PUQE score (Q2-Q1)		
				Mean change (SD)	Crude difference in mean changes, β (95% CI)	Adjusted difference in mean changes^b^, β (95% CI)
**Primary analysis**						
	Intervention group (n=88)	4.9 (2.0)	5.6 (1.8)^c^	0.8 (2.0)	0.4 (−0.3 to 1.2)	0.6 (−0.1 to 1.2)
	Control group (n=103)	4.7 (1.9)	4.9 (1.8)^d^	0.4 (2.3)	Reference	Reference
**Subanalyses by employment: women employed in the health sector**						
	Intervention group (n=19)	4.6 (1.9)	6.6 (1.7)^e^	1.8 (2.5)	2.1 (0.3 to 3.9)	2.1 (0.9 to 3.2)
	Control group (n=31)	4.5 (1.9)	4.6 (1.6)^f^	−0.3 (2.7)	Reference	Reference
**Subanalyses by employment: women employed in other sectors**						
	Intervention group (n=55)	4.9 (2.1)	5.2 (1.7)^g^	0.4 (1.7)	−0.1 (−0.8 to 0.7)	0.0 (−0.7 to 0.7)
	Control group (n=60)	4.7 (1.9)	5.1 (1.8)^h^	0.5 (1.9)	Reference	Reference

^a^This score ranges from 3 to 15 points, and symptoms are rated as follows: mild: ≤6 points; moderate: 7-12 points; severe ≥13 points.

^b^Adjusted for the baseline PUQE score.

^c^n=59.

^d^n=78.

^e^n=14.

^f^n=23.

^g^n=38.

^h^n=45.

### Impact on Quality of Life

The adjusted primary analysis showed that the changes in NVPQOL scores from baseline to Q2 were not significantly different between the intervention and control groups (adjusted β −5.3, 95% Cl −12.5 to 1.9; Table 3).

**Table 3:** Association between the use of the MinSafeStart (MSS) app and quality of life.

Group	Baseline (Q1) NVPQOL^a,b^ score, mean (SD)	Follow-up (Q2) NVPQOL score, mean (SD)	Change in NVPQOL score (Q2-Q1)		
			Mean change (SD)	Crude difference in mean changes, β (95% CI)	Adjusted difference in mean changes^c^, β (95% CI)
Intervention group (n=88)	145.7 (34.0)	143.8 (29.7)^d^	−4.5 (22.4)	−4.2 (−11.9 to 3.5)	−5.3 (−12.5 to 1.9)
Control group (n=103)	148.5 (28.8)	151.6 (28.9)^e^	−0.3 (22.9)	Reference	Reference

^a^NVPQOL: Health-Related Quality of Life for Nausea and Vomiting during Pregnancy scale.

^b^This score ranges from 30 to 210 points, and lower scores indicate better quality of life.

^c^Adjusted for the baseline NVPQOL score.

^d^n=59.

^e^n=78.

### Impact on Decisional Conflict Scale Score

The mean changes in the DCS between Q1 and Q2 were −5.9 (SD 16.4) for the intervention group and −5.3 (SD 15.5) for the control group (Table 4). The changes in DCS were not significantly different between the women in the intervention group and the women in the control group (adjusted β −1.1, 95% Cl −6.2 to 4.2).

**Table 4:** Association between the use of the MinSafeStart (MSS) app and the decisional conflict scale (DCS).

Group	Baseline (Q1) DCS, mean (SD)		Follow-up (Q2) DCS, mean (SD)		Change in DCS^a^ (Q2-Q1)				
					Mean change (SD)		Crude difference in mean changes, β (95% CI)		Adjusted difference in mean changes^b^, β (95% CI)
Intervention group (n=88)	88	40.3 (17.9)	36.2 (21.6)^c^	−5.9 (16.4)		−0.7 (−6.1 to 4.7)		−1.1 (−6.2 to 4.2)	
Control group (n=103)	103	42.5 (20.9)	38.1 (20.3)^d^	−5.3 (15.5)		Reference		Reference	

^a^This score ranges from 0 points (no decisional conflict) to 100 points (extremely high decisional conflict).

^b^Adjusted for the baseline decisional conflict score.

^c^n=59.

^d^n=78.

### Association Between NVP Severity and Quality of Life

**Figure 4:**
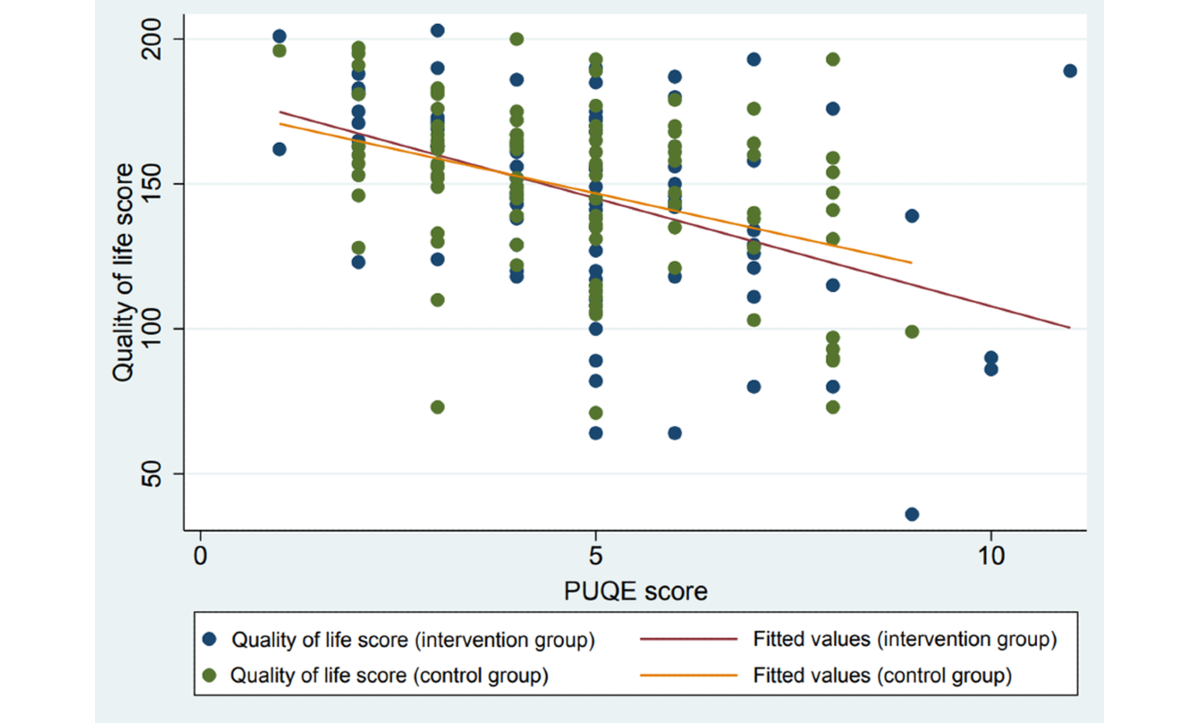
Association between the Health-Related Quality of Life for Nausea and Vomiting during Pregnancy score (NVPQOL) score and the Pregnancy Unique Quantification of Emesis (PUQE) score. MSS app: MinSafeStart mobile application.

Women with more severe NVP (higher PUQE scores) had lower NVPQOL scores than women with less severe NVP (lower PUQE scores; Figure 4).

## Discussion

### Main Findings

The MinSafeStart trial was the first to investigate the effectiveness of a patient-centered mobile app that was designed to empower pregnant women to optimally manage their NVP symptoms. We found no significant associations between the use of the MSS app and the severity of NVP symptoms, QoL, or decisional conflict, compared with standard maternal care. These results should be interpreted with caution because the study was slightly underpowered, due to a higher dropout rate than expected.

Earlier studies have shown that the majority of the pregnant population owns a smartphone and over 50% use apps related to pregnancy [36]. Studies that have investigated the use of health-related apps have shown that the apps could improve the knowledge levels of pregnant women and the apps were perceived as tools during pregnancy [7,24]. Except for user satisfaction, our results were not consistent with those from previous studies. We found no associations between the use of the MSS app and NVP symptoms at 2 weeks after baseline. This may be explained by several factors related to our study population and study design. First, we included women at any gestational stage in pregnancy. In fact, 15% of the women included were beyond the first trimester, which is the most relevant time window for NVP. On average, NVP occurs during gestational week 4 [10] and peaks during gestational weeks 10-16 [37,38]. However, our intervention group had completed a median of 8 gestational weeks at enrollment, with a range of 4-36 weeks. Therefore, in many cases, it may have been too late for women to benefit from the app. Moreover, we included women with mild NVP, and this group may not derive the most benefit from the app. Second, a 2-week follow-up may not have been optimal for evaluating the effect of the intervention. The rationale for choosing a 2-week follow-up was based on earlier studies that showed that PUQE scores decreased by 4.7 points when treated within 1 week [39]. We could not exclude the possibility that natural fluctuations in NVP severity could have affected the results or that a shorter follow-up time before the app assessment might have been a better choice. In fact, there might not be a particular time that is optimal for measuring the effects of the app. Indeed, NVP severity varies from morning to evening and from day to day. Therefore, selecting a specific time point for follow-up and reporting the PUQE score in Q2 may not have fully captured the changes in NVP severity over time. Future studies should consider these elements when designing a trial to evaluate the effect of using a digital tool during pregnancy.

Another factor that may have affected the results was that the study included a high proportion of parous women with a prior NVP history. Moreover, most were in a relationship with a partner, which may have provided emotional support. Therefore, these women may have already been informed about optimal NVP management and treatment, and consequently, they may not have felt they needed more information from an NVP tool. Many earlier studies have shown that women with a higher sociodemographic status and women who are pregnant for the first time are more likely to search for information online [40-42]. In their first pregnancy, women often search for information about concerns and symptoms related to the first period of pregnancy [6,40,43-45]. Therefore, our study may not have targeted the appropriate subgroup of pregnant women.

### Strengths and Limitations

The main strength of this study was that very few studies have been conducted to assess the effectiveness of mobile apps for disease management among pregnant women. This study provided new insights in this regard. An important strength of this study was the use of the randomized controlled trial study design, which is considered the gold standard in evidence-based medicine [46]. Another strength of this study included our use of the internet for recruitment and electronic data collection. The main benefit of social media recruiting is that it is convenient for sampling. Indeed, pregnant women in their first trimester are not given any routine care, and there is no ideal place to reach out to this group, outside of social media. This approach facilitated the participation of pregnant women all over Norway, which may have increased the representativeness of the study sample and, thus, the generalizability of the results. In addition, the NVPQOL may have provided an advantage over other QoL scales because the NVPQOL is more specific [40].

The major limitation of this study was that we did not reach our targeted number of participants, which was 250 women, including a 25% dropout rate. Furthermore, as in all studies based on voluntary patient recruitment, there might have been a self-selection bias, where more motivated and resourceful women are included in the study compared with the general population. Participants who were parous women with higher sociodemographic status than the general birthing population in Norway might also have contributed to a selection bias. Because these women might have been more informed about optimal NVP management, they might have had less use for the app. We could not exclude the possibility that this selection bias might explain why we did not find any significant beneficial effect of the app on NVP severity in this study.

Last, 15% of the women in the intervention group were beyond the first trimester when the app was introduced. It may have been too late for many of these women to take advantage of the app because NVP often occurs in week 4 [10] and it peaks around weeks 10-16 [37,38].

### Future Research

Digitalization and eHealth have provided opportunities to develop innovative apps that support pregnant women. These mobile applications must be tested in clinical studies to establish evidence for health efficacy before they can be included in the health care system or recommended by health care personnel [47]. Our review from 2020, consistent with previous studies [48], demonstrated that decision support tools could potentially provide benefit to pregnant women. However, the tools were mainly useful when relevant information was assembled into one digital tool and when the woman could share her recordings with her health care provider [7]. Based on the results of this study, future research should focus on how to design trials to determine the effect of digital tools on the pregnancy outcomes that are most important to pregnant patients. Future studies should also investigate whether digital tools and apps might be more effective when developed as part of a more extensive health intervention. Specific focus should be placed on how digital tools might facilitate counseling and communication between pregnant women and health care providers regarding NVP management in pregnancy.

### Conclusion

This study showed that tracking NVP symptoms with a mobile application was not associated with reduced NVP symptoms, less decisional conflict, or improved QoL after 2 weeks of use. These findings may have been influenced by study design–related factors, such as the gestational week of enrollment, women’s parity, time to follow-up, and sample size. Future studies should include a process evaluation to improve our understanding of how pregnant women use the app and how to optimize its utility within maternity care.

## Appendix

Multimedia Appendix 1 Management of nausea and vomiting in pregnancy (NVP), according to treatment guidelines. PUQE= Pregnancy Unique Quantification of Emesis score; this score ranges from 3 to 15 points.

Multimedia Appendix 2 The questions in the PUQE score, NVPQOL scale, and the decisional conflict scale. PUQE= Pregnancy Unique Quantification of Emesis; NVPQOL=.

Multimedia Appendix 3 CONSORT-eHEALTH checklist (V 1.6.1)

## Abbreviations

DCS: decisional conflict scale
HG: hyperemesis gravidarum
MSS: MinSafeStart
NVP: nausea and vomiting in pregnancy
NVPQOL: Health-Related Quality of Life for Nausea and Vomiting during Pregnancy scale
PUQE: Pregnancy Unique Quantification of Emesis
Q1: Questionnaire 1
Q2: Questionnaire 2
Q3: Questionnaire 3
Q4: Questionnaire 4
QoL: quality of life
TSD: Service for Sensitive Data at the University of Oslo
USIT: University Center for Information Technology
