# Authorship Correction: Impact of a Mobile Application for Tracking Nausea and Vomiting During Pregnancy (NVP) on NVP Symptoms, Quality of Life, and Decisional Conflict Regarding NVP Treatments: MinSafeStart Randomized Controlled Trial

**DOI:** 10.2196/41927

**Published:** 2022-09-26

**Authors:** Elin Ngo, Maria Bich-Thuy Truong, David Wright, Hedvig Nordeng

**Affiliations:** 1 PharmacoEpidemiology and Drug Safety Research Group Department of Pharmacy University of Oslo Oslo Norway; 2 School of Allied Health Professions University of Leicester England United Kingdom; 3 Centre for Pharmacy University of Bergen Bergen Norway; 4 Department of Child Health and Development National Institute of Public Health Oslo Norway

In “Impact of a Mobile Application for Tracking Nausea and Vomiting During Pregnancy (NVP) On NVP Symptoms, Quality of Life, and Decisional Conflict Regarding NVP Treatments: MinSafeStart Randomized Controlled Trial” (JMIR Mhealth Uhealth 2022;10(7):e36226) the authors noted one error.

 In the originally published article, authors Hedvig Nordeng and David Wright were ordered as, respectively, third and fourth authors. The correct order of authors should be David Wright as third author and then Hedvig Nordeng as fourth author. Author affiliations have been reordered accordingly.

The complete list of authors and affiliations in the originally published article was as follows:

Elin Ngo^1^, MSc; Maria Bich-Thuy Truong^1^, PhD; Hedvig Nordeng^1,2^, PhD; David Wright^3,4^, PhD

^1^PharmacoEpidemiology and Drug Safety Research Group, Department of Pharmacy, University of Oslo, Oslo, Norway

^2^Department of Child Health and Development, National Institute of Public Health, Oslo, Norway

^3^School of Allied Health Professions, University of Leicester, England, United Kingdom

^4^Centre for Pharmacy, University of Bergen, Bergen, Norway

The list of authors and affiliations has been corrected to:

Elin Ngo^1^, MSc; Maria Bich-Thuy Truong^1^, PhD; David Wright^2,3^, PhD; Hedvig Nordeng^1,4^, PhD

^1^PharmacoEpidemiology and Drug Safety Research Group, Department of Pharmacy, University of Oslo, Oslo, Norway

^2^School of Allied Health Professions, University of Leicester, England, United Kingdom

^3^Centre for Pharmacy, University of Bergen, Bergen, Norway

^4^Department of Child Health and Development, National Institute of Public Health, Oslo, Norway

The correction will appear in the online version of the paper on the JMIR Publications website on September 26, 2022, together with the publication of this correction notice. Because this was made after submission to PubMed, PubMed Central, and other full-text repositories, the corrected article has also been resubmitted to those repositories.

