# Supplementary material for: Impact of a Mobile Application for Tracking Nausea and Vomiting During Pregnancy (NVP) on NVP Symptoms, Quality of Life, and Decisional Conflict Regarding NVP Treatments: MinSafeStart Randomized Controlled Trial
Source: JMIR Mhealth Uhealth. 2022 Jul 5;10(7):e36226. doi: 10.2196/36226 (PMC9297140; doi:10.2196/36226)
Supplement: Multimedia Appendix 1 [file mhealth_v10i7e36226_app1.docx]

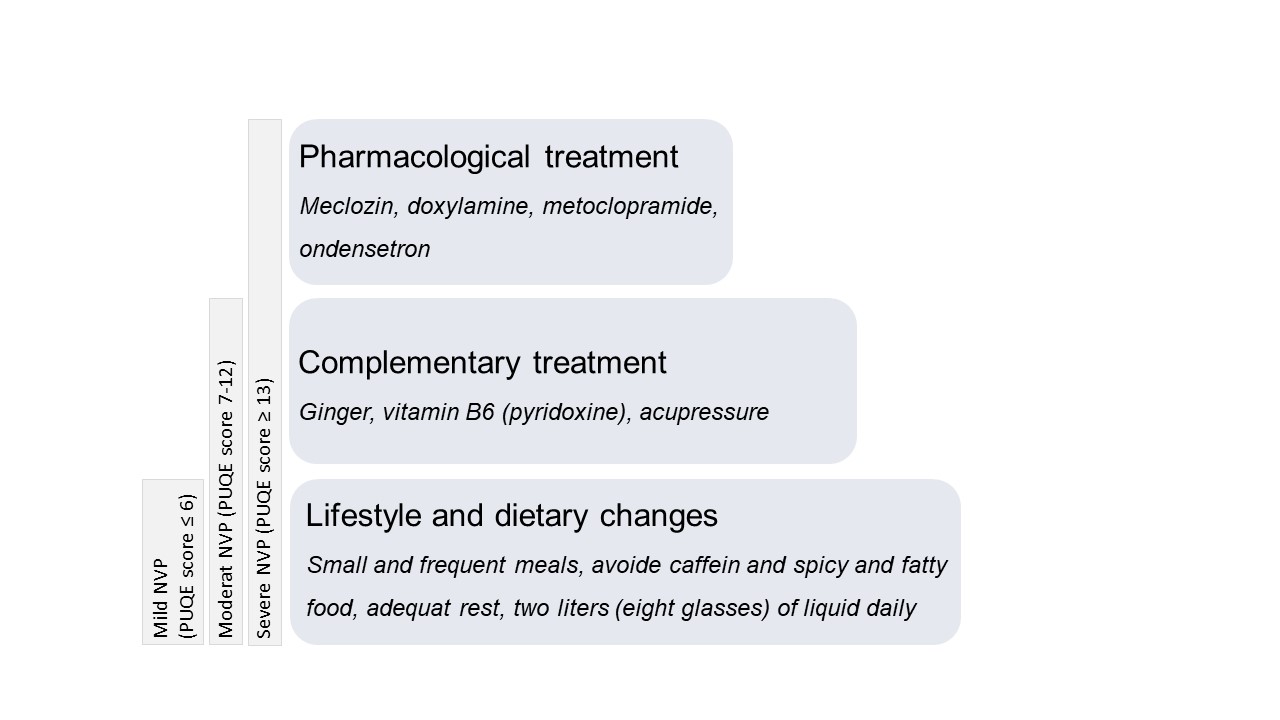


**Multimedia appendix 1:** Management of nausea and vomiting in pregnancy (NVP), according to treatment guidelines. PUQE= Pregnancy Unique Quantification of Emesis score; this score ranges from 3 to 15 points**.**
