# Supplementary material for: Impact of a Mobile Application for Tracking Nausea and Vomiting During Pregnancy (NVP) on NVP Symptoms, Quality of Life, and Decisional Conflict Regarding NVP Treatments: MinSafeStart Randomized Controlled Trial
Source: JMIR Mhealth Uhealth. 2022 Jul 5;10(7):e36226. doi: 10.2196/36226 (PMC9297140; doi:10.2196/36226)
Supplement: Multimedia Appendix 2 [file mhealth_v10i7e36226_app2.docx]

**Multimedia appendix 2:** The questions in the PUQE score, NVPQOL scale, and the Decisional conflict scale.

**Pregnancy-Unique Quantification of Emesis and Nausea (PUQE) score**

*Answer the option that suits the best for your situation for the last 24 hours.*

| 1 | On average in a day, for how long do you feel nauseated or sick to your stomach? | | | | | |
| --- | --- | --- | --- | --- | --- | --- |
| Answer option | > 6 hours | | 4-6 hours | 2-3 hours | ≤ 1 hour | Not at all |
| 2 | On average in a day, how many times do you vomit or throw up? | | | | | |
| Answer option | > 6 hours | | 4-6 hours | 2-3 hours | ≤ 1 hour | Not at all |
| 3 | On average in a day, how many times have you had retching or dry heaves without brining anything up? | | | | | |
| Answer option | > 6 hours | 4-6 hours | | 2-3 hours | ≤ 1 hour | Not at all |
| 4 | On a scale of 0 to 10, how would you rate your well-being: _________ 0 (worst possible) 10 (as good as you felt before pregnancy) | | | | | |

**Nausea and vomiting in pregnancy Quality of life (NVPQOL)**

Over the past week, from 1 (none of the time) to 7 (all of the time) how much have you been experiencing…

| 1. Nausea |
| --- |
| 2. Feeling sick to your stomach |
| 3. Vomiting |
| 4. Dry-heaves (vomiting without bringing anything up) |
| 5. Poor Appetite |
| 6. Symptoms being worse in the evening |
| 7. Not eating for longer than you would like |
| 8. Feeling worse when exposed to certain smells |
| 9. Feeling worse when exposed to certain foods |
| 10. Fatigue |
| 11. Feeling worn-out and loss of energy |
| 12. Feeling exhausted |
| 13. Feeling tired |
| 14. Feeling emotional |
| 15. Being less interested in sex |
| 16. Feeling downhearted, blue, sad, unhappy, depressed, gloomy |
| 17. Feeling frustrated |
| 18. Feeling fed up with being sick |
| 19. Not feeling that your symptoms are all part of normal pregnancy |
| 20. Feeling that you can`t enjoy your pregnancy |
| 21. That everything is an effort |
| 22. Feeling like you have accomplished less than you would like |
| 23. That it takes longer to get things done that usual |
| 24. Difficultly performing your work and activities |
| 25. Difficultly maintaining your normal social activities |
| 26. Relying on your partner for doing things that you would normally do |
| 27. Difficulty looking after your home |
| 28. Difficulty shopping for food |
| 29. Difficulty preparing or cooking meals |
| 30. Cutting down on amount of time you spend at work or other activities |

**Decisional Conflict Scale (DCS)**

*Which treatment option do you prefer for nausea and vomiting during pregnancy?*

*Please check one.*

1. Self-care
2. Antiemetic drugs
3. Self-care and antiemetic drugs

*Considering the option you prefer (strongly agree, agree, neither agree or disagree, disagree, strongly disagree), please answer the following questions:*

| **Statement** |
| --- |
| I know which options are available to me. |
| I know the benefits of each option. |
| I know the risk and side effects of each option. |
| I am clear about which benefits matter most to me. |
| I am clear about which risks and side effects matter most. |
| I am clear about which is more important to me (the benefits or the risks and side effects). |
| I have enough support from others to make a choice. |
| I am choosing without pressure from others. |
| I have enough advice to make a choice. |
| I am clear about the best choice from me. |
| I feel sure about what to choose. |
| This decision is easy for me to make. |
| I feel I have made an informed choice. |
| My decision shows what is important to me. |
| I expect to stick with my decision. |
| I am satisfied with my decision. |
